# Fatigue and recovery assessed by repetitive handgrip strength measurement as predictors of fall risk in older adults: A cross-sectional study

**DOI:** 10.1177/02692155251355881

**Published:** 2025-06-30

**Authors:** Ali Kapan, Milos Ristic, Richard Felsinger, Thomas Waldhoer

**Affiliations:** 1 Center for Public Health, Department of Social and Preventive Medicine, 27271 Medical University of Vienna, Vienna, Austria; 2 Center for Public Health, Department of Epidemiology, 27271 Medical University of Vienna, Vienna, Austria

**Keywords:** Muscle fatigue, rehabilitation, handgrip, assessment

## Abstract

**Design:**

A cross-sectional study assessing the utility of repetitive handgrip strength measurements for predicting fall risk in older adults.

**Setting:**

Conducted in two residential care homes in Vienna, Austria.

**Participants:**

217 older adults (mean age: 80.2 years, 65.9% female) participated. Those with significant cognitive impairments Mini-Mental State Examination ≤17, severe neuromuscular disorders, or recent hand injuries were excluded.

**Intervention:**

Participants underwent a repetitive handgrip strength protocol, comprising 10 maximal grips (3-second contractions, 5-second rest intervals) performed twice, with a one-hour interval. Fatigability and recovery ratios were calculated. Functional assessments included the Short Physical Performance Battery, the Physical Activity Scale for the Elderly, the Falls Efficacy Scale-International, and the Multidimensional Fatigue Inventory. Retrospective and prospective fall data were also collected.

**Main Measures:**

Handgrip strength parameters (maximum, fatigue ratio, recovery ratio) and fall events (retrospective and prospective) were analysed alongside physical and functional assessments.

**Results:**

Fallers (39.6%) had higher fatigue ratios (median: 1.4 vs. 1.2) and lower recovery ratios (median: 0.9 vs. 1.0) compared to non-fallers (*P* < .001). Fatigue ratio was a strong predictor of prospective falls (incidence rate ratio: 1.14, 95% confidence interval: 1.09–1.41). Recovery ratio showed marginal significance. Functional measures were also strongly associated with fall risk.

**Conclusions:**

Repetitive maximum handgrip strength measurements dynamically assess neuromuscular performance and offer superior predictive power for fall risk compared to standard single maximum handgrip strength. Fatigue and recovery ratios should be incorporated into fall risk assessment to improve prevention strategies in older adults.

## Background

Muscle fatigability plays a crucial role in the risk of falls in older adults, which has become a major public health issue worldwide.^1^ Both lower and upper limb fatigability influence fall biomechanics.^2,3^ Studies have shown different patterns of fatigability: general and central fatigability dominate in the lower limbs, while peripheral fatigability and corticospinal inhibition are more prominent in the upper limbs. Consequently, measuring handgrip strength may provide a more targeted assessment of recovery capacity following muscle exertion.^4^ The importance of assessing muscle fatigability through handgrip strength has been demonstrated in various conditions characterised by altered muscle function. In recent years, this approach has proven valuable in studying individuals with Long COVID-19 and Myalgic Encephalomyelitis/Chronic Fatigue Syndrome.^5–7^ The mechanisms of muscle fatigability in these conditions share similarities with age-related changes, particularly regarding mitochondrial dysfunction and inflammatory processes.^8,9^

While maximum handgrip strength is an effective indicator of sarcopenia and frailty, single measurements cannot assess fatigability or recovery capacity.^10,11^ However, fatigability and recovery capacity have been shown to be better predictors of daily performance in older adults than single maximum handgrip strength alone.^12,13^ These factors are also crucial for fall prevention, as even individuals with normal maximum handgrip strength may have an increased risk of falling.^14^ Maximum handgrip strength only measures peak force production. It does not capture other important factors like muscular endurance, fatigability, or functional capacity in daily activities. Consequently, maximum handgrip strength may be an incomplete measure of overall muscle performance and should not be relied upon alone to assess muscle weakness.^15,16^ Furthermore, fatigue/exhaustion is often assessed as a single question in self-report instruments such as the Fried phenotype.^17^ Studies have shown that objective methods of quantifying neuromuscular fatigability provide a more accurate assessment of fatigability and its effects, and can detect changes in muscle performance and recovery before they are subjectively perceived.^18,19^ To date, no study has examined whether fatigue and recovery parameters derived from repetitive handgrip strength testing are associated with fall risk in older adults. We therefore developed a protocol using 10 consecutive maximal handgrip repetitions to capture both neuromuscular fatigability and recovery capacity beyond what is measurable through single maximal force or self-report. The aim of this study is to explore the relationship between repetitive handgrip strength measures, muscle fatigability and recovery capacity in older adults, particularly concerning fall risk. Falls are a leading cause of death and injury in people over the age of 65. Accurate assessment of fall risk is therefore essential for prevention.^20^ This study examines how both single maximal and repetitive handgrip strength measurements, along with fatigue ratios and recovery parameters, relate to fall risk in older adults.

## Methods

### Study design and participants

This study is a secondary analysis of data from a cross-sectional study carried out between January and July 2024 in two residential care homes in Vienna.^21^ Using a convenience sampling approach, we recruited participants from these facilities. The study was carried out in accordance with the principles set out in the Declaration of Helsinki and in accordance with the strengthening the Reporting of Observational Studies in Epidemiology guidelines. Ethical approval was obtained from the Clinical Research Ethics Board of Vienna (EK-23-082-0523). To be included in the study, people had to be able to walk independently or use assistive devices, give informed consent, and have a basic understanding of German or English. Individuals with cognitive impairment, defined by a Mini Mental State Examination score ≤17, were excluded due to potential difficulties in following instructions.^22^ In addition, individuals with conditions that affect hand strength, such as severe rheumatoid arthritis, carpal tunnel syndrome, advanced osteoarthritis, paralysis, stroke sequelae, recent hand injury, or severe neuromuscular disorders, were excluded to avoid confounding the handgrip strength measurements.

### Procedure and measurements

Two physiotherapists, each with over 5 years’ experience in geriatric and rehabilitation assessment, carried out the measurements. To guarantee validity and objectivity, participants were randomly assigned to the two physiotherapists using a web-based randomisation tool from the Medical University of Vienna (https://www.meduniwien.ac.at/randomizer/). A member of the research team, who did not participate in the assessments, created the randomisation sequence before data collection began. The assessments were conducted in the morning in 2 identical rooms with standardised flooring, lighting, and room size. Several physical performance tests were administered at the beginning of the assessment. The assessment included the Short Physical Performance Battery, which evaluates lower limb function through balance, gait speed, and chair-stand tests,^23^ and a standardised maximum handgrip strength.^24^ The maximum handgrip strength test consisted of 3 repetitions with 1–2 minutes rest between each repetition, following standard procedures commonly used in studies. After the physical tests, participants completed several questionnaires, including the Physical Activity Scale for the Elderly^25^ for weekly activity levels, the Multidimensional Fatigue Inventory^26^ for subjective fatigue across various domains, the Athens Insomnia Scale^27^ for sleep disturbances, and the Falls Efficacy Scale-International^28^ to assess fear of falling during daily activities. This sequence was chosen to allow sufficient recovery time between the different handgrip strength measures. Following the questionnaires, participants performed the first repetitive handgrip strength test. For each repetition, they squeeze the handgrip with maximum force for 3 seconds, followed by a 5-second rest. This sequence was repeated 10 times in the same way, with an interval timer to ensure accurate timing (Round 1). One hour later (Round 2), the same 10 3-second handgrip repetitions were performed again to assess fatigability from the previous tests and to evaluate the participants’ state of recovery.

By including, established assessment tools (e.g. Short Physical Performance Battery, Physical Activity Scale for the Elderly, the Multidimensional Fatigue Inventory, and the Falls Efficacy Scale-International) alongside measures of repetitive handgrip strength – which capture physical factors such as muscle strength, fatigability and recovery capacity – this study allows a detailed examination of their relationship with fall risk in older adults. Each variable addresses specific aspects of physical and psychological functioning. Handgrip strength is measured as described above using a digital hand dynamometer (CAMRY, model: SCACAM-EH101) according to standardised procedures.^29^ Hand dominance was determined through self-report, with participants indicating their preferred hand for writing and daily activities. Participants sit in a neutral position with their elbows bent at a 90-degree angle. For the standard maximum handgrip strength measurement, they press the dynamometer as hard as possible for 3 seconds, with three trials per hand and a 2-minute rest between trials. The highest values were recorded. For the repetitive handgrip strength measurement, the maximum force is applied to the dynamometer 10 times in succession for 3 seconds each, using the dominant hand, with a 5-second rest between trials. Table 1 summarises the measurements and calculations made to evaluate the handgrip strength and associated fatigability and recovery parameters.

**Table 1.** Muscle strength, fatigue, and recovery parameters assessed by repetitive handgrip strength measurement.

Parameter/Ratio	Formula	Explanation
Fmax 1, 2 (Round 1 and Round 2)	Fmax [kg]	Maximum handgrip strength within one session (10 repeat trials)
Fmean 1, 2 (Round 1 and Round 2)	∑10pulls/10	Mean handgrip strength of all 10 trials
Fatigue ratio 1, 2 (assessment of fatigability) (Round 1 and Round 2)	Fmax/Fmean	Higher values indicate a stronger decrease in force during one session
Recovery ratio (assessment of recoverability)	Fmean2/Fmean1	A higher value is an indication of better recovery after two measurements.

To ensure valid and comprehensive reporting of falls, data was collected from available medical records. The recording of falls was facilitated by the Cogvis 3D sensor system, which is wall- or ceiling-mounted and can automatically detect falls without the need for residents to wear a device. In addition, the system has a preventative function, such as automatically turning on lights when residents get up at night to prevent falls. The system has been validated in an extensive field study evaluating over 2.2 million events in more than 1200 installations, demonstrating high fall detection accuracy while maintaining privacy.^30^ Participants were also asked directly whether and how often they had fallen in the previous 12 months and 4 months after the measurement. A fall was defined as an event in which a person unintentionally falls to the ground, floor or one level below. This definition excludes falls caused by external force or medical events such as stroke or seizure.^31^

The following characteristics were obtained from the medical records: age, sex, body mass index, comorbidities as measured by the Charlson Comorbidity Index, Barthel Index, Mini Mental State Examination and current medication.

### Sample size

A formal a priori sample size calculation was not carried out, as the study aimed to include all eligible residents from two large residential care facilities in Vienna. The final sample size of 217 participants represents the total number of residents who met the inclusion criteria and consented to participate.

## Statistics

Descriptive statistics were used to summarise the demographic characteristics of the participants, calculating the mean and standard deviation for continuous variables and using frequencies and percentages for categorical variables. Data distribution was assessed using the Shapiro–Wilk test and through visual inspection of histograms to evaluate the assumption of normality. Parametric tests were applied to normally distributed variables, while non-parametric tests were utilised when this assumption was not met. Paired *t*-tests were used to compare measurements within each time point (initial and 1 hour later), a Friedman test was used for repeated measurements within each group (Fmax standard, Fmax1, Fmax2) and a Kruskal-Wallis test was used to assess differences between the non-fallers and fallers groups. Pearson and Spearman rank correlation coefficients were calculated to determine linear and non-linear correlations, respectively, between all potential predictors (Falls Efficacy Scale-International, Physical Activity Scale for the Elderly, Short Physical Performance Battery, Multidimensional Fatigue Inventory, Athens Insomnia Scale, standard maximum handgrip strength, fatigue and recovery ratios) with fall events.

Multicollinearity diagnostics were performed prior to the main analysis. Variance inflation factors (VIF) were used to assess the presence of multicollinearity, with a threshold of >2.5. Fatigue Ratio 2 had a VIF of 4.2, exceeding the threshold and indicating moderate multicollinearity. With the exception of Fatigue Ratio 2, all other VIF values were below the threshold. Consequently, Fatigue Ratio 2 was excluded from further analysis to mitigate potential multicollinearity issues. Finally, given the count nature of the dependent variables (number of falls) and the overdispersion observed (dispersion parameter > 1.5), a negative binomial model was implemented. The model was estimated using the GENLIN procedure, with the selection of the model based on the assessment of its goodness of fit, the Akaike information criterion and the Bayesian information criterion. Two separate analyses were performed to examine the relationship between health and function-related variables and fatigue and recovery ratios with the number of falls: a prospective analysis with falls in the 4 months after the first assessment as the dependent variable, and a retrospective analysis with falls in the 12 months before the first assessment as the dependent variable. Both analyses used identical sets of independent variables. In addition, in the prospective analysis, the number of retrospective falls in the previous 12 months was included as an independent variable to account for the influence of previous fall events on future fall risk. To quantify the associations, univariate and multivariable incidence rate ratios with 95% confidence intervals were calculated for both periods. Variables that reached statistical significance in the univariate analysis were included in the multivariate models. Model 1 was adjusted for age and sex, while Model 2 additionally controlled for potential confounding variables, including Charlson Comorbidity Index, Barthel Index, Mini Mental State Examination and number of medication. Given the exploratory nature of the study, *P*-values were not adjusted for multiple testing, and this should be taken into account when interpreting the results. All statistical analyses were performed using IBM SPSS Statistics software, version 27.0.

## Result

Out of 281 residents evaluated for eligibility, 217 were included in the study. Among these, 131 participants (60.4%) were identified as non-fallers, while 86 participants (39.6%) were categorised as fallers based on their fall history from the previous 12 months before the initial assessment. Of the 86 fallers, 40 individuals (46.5%) had experienced 1 fall, 37 individuals (43.0%) had experienced 2 falls and 9 individuals (10.5%) had experienced 3 falls. The mean age of the participants was 80.2 years (standard deviation 4.3), while that of the non-fallers was 78.3 years (standard deviation 3.7), younger than that of the fallers, who had a mean age of 83.4 years (standard deviation 2.9). In terms of sex distribution, 65.9% (143 participants) were female, with a higher proportion of females in the fallers group (88.4%), whereas almost half of the non-fallers group (49.9%) were male. In the 4 months following the baseline assessment, 58 (67.4%) of the fallers experienced a fall, while only 3 (2.3%) of the non-fallers experienced a fall in the same period (Table 2).

**Table 2.** Demographic and physical characteristics of participants by fall status in the past 12 months.

Variables	Total (*n* = 217)	Non-fallers (*n* = 131)	Fallers (*n* = 86)
Age, mean (SD)	80.2 (4.3)	78.3 (3.7)	83.4 (2.9)
Sex *n* (%)			
Female	143 (65.9)	67 (51.1)	76 (88.4)
Male	74 (34.1)	64 (49.9)	10 (11.6)
BMI, mean (SD)	24.3 (2.9)	24.6 (2.7)	23.9 (3.1)
Barthel Index	82.4 (12.7)	89.2 (12.5)	72.1 (12.6)
Number of medication, median (min–max)	7 (6–14)	6 (6–12)	9 (6–14)
Charlson comorbidity, mean (SD)	2.3 (1.2)	1.8 (0.9)	3.1 (1.1)
MMSE Score, mean (SD)	26.3 (2.8)	28.1 (1.6)	24.2 (2.2)
SPPB Score, mean (SD)	8.3 (2.8)	9.6 (2.4)	6.2 (2.1)
FES-I Score, mean (SD)	28.8 (13.2)	22.7 (11.4)	38.1 (10.1)
PASE Score, mean (SD)	79.5 (44.7)	89.4 (48.2)	52.1 (26.6)
AIS Score mean (SD)	6.9 (3.2)	5.8 (2.4)	7.4 (2.8)
MFI Score, mean (SD)	49.4 (8.4)	46.2 (9.7)	53.6 (7.7)
Standard maximum handgrip strength, mean (SD)	21.9 (7.7)	25.9 (6.4)	15.8 (4.8)
Falls in the last 12 months, *n* (%)			
No Fall	131 (60.4)	0	131 (60.4)
1 Fall	35 (16.1)	0	35 (16.1)
2 Fall	32 (14.7)	0	32 (14.7)
3 Fall	16 (7.4)	0	16 (7.4)
≥4 Falls	3 (1.4)	0	3 (1.4)
Falls within 4 months after survey, *n* (%)			
No Fall	161 (74.2)	121 (92.4)	40 (46.5)
1 Fall	38 (17.5)	9 (6.9)	29 (33.7)
2 Fall	14 (6.5)	1 (0.7)	13 (15.1)
3 Fall	4 (1.8)	0	4 (4.7)

Note: SD = Standard deviation, BMI = Body Mass Index, AIS = Athens Insomnia Scale, MMSE = Mini Mental State Examination, PASE = Physical Activity Scale for the Elderly, MFI = Multidimensional Fatigue Inventory, SPPB = Short Physical Performance Battery, maximum handgrip strength from three rounds.

**Figure 1.**
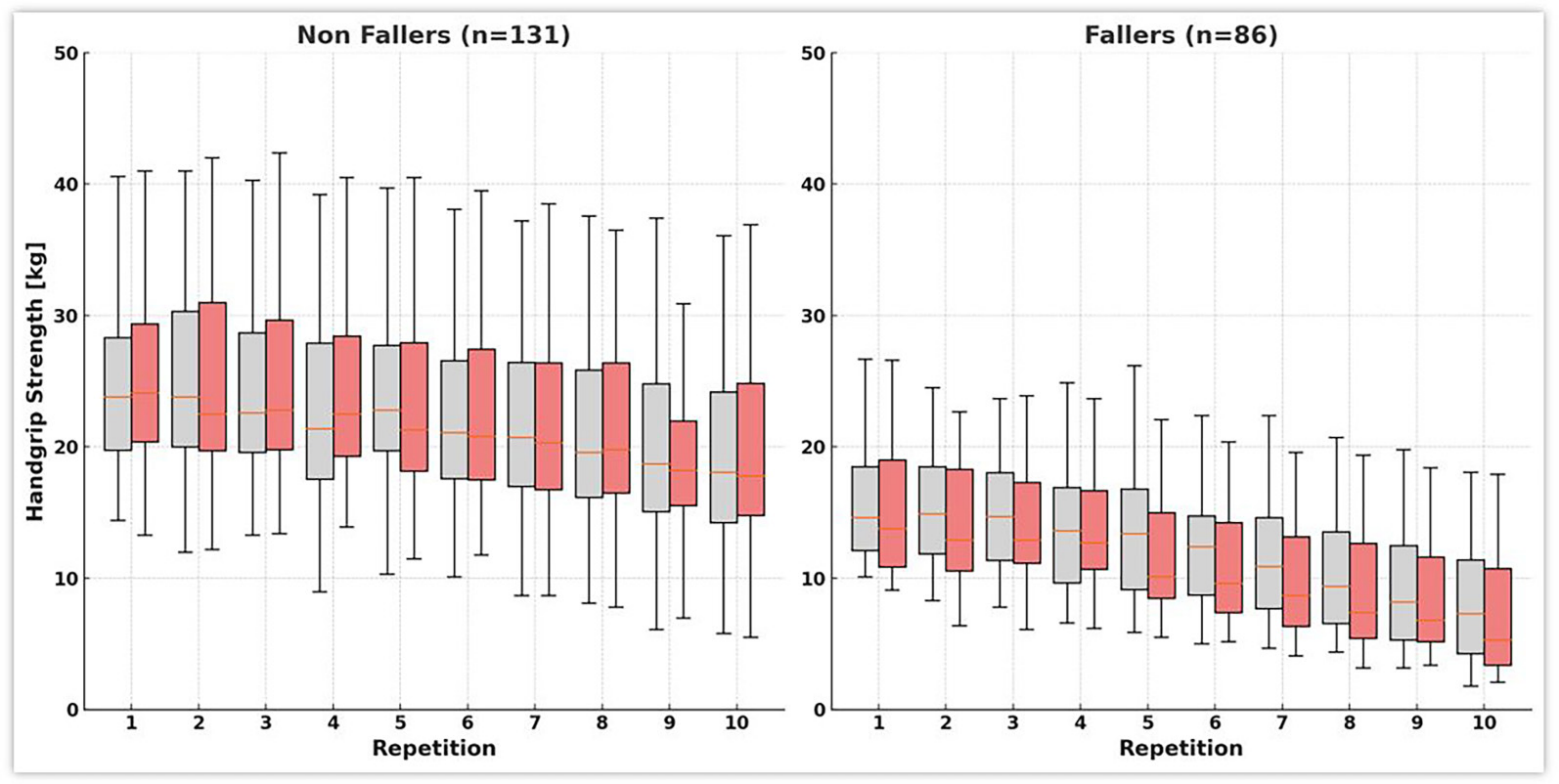
The box plots display grip strength (kg) over 10 repetitions for two independent measurement rounds in each group. Boxes with lighter fill indicate round 1, and boxes with darker fill indicate round 2. Each box shows the median (central line) and interquartile range (Q1–Q3) without outliers; whiskers denote the range of the remaining observations. Some participants were unable to complete all repetitions.

All 217 participants completed the standard maximum handgrip strength measurement, followed by 10 initial repetitions and a repeat test after 60 minutes. Table 3 and Figure 1 present the Fmax and Fmean values, as well as fatigue and recovery rates, for both fallers and non-fallers. Non-fallers had a median Fmax standard of 24.6 kg (interquartile range (IQR): 22.3–31.1) and an Fmax 1 of 24.1 kg (IQR: 21.2–30.9), which increased significantly to 25.4 kg (IQR: 21.7–32.1) after 1 hour (Fmax 2). In contrast, fallers had a median Fmax standard of 14.9 kg (IQR: 12.1–19.7), with a median Fmax 1 of 15.4 kg (IQR: 12.7–19.8), which decreased to a median of 13.8 kg (IQR: 11.8–19.0) in Fmax 2. For Fmean, non-fallers showed minimal changes with a median Fmean1 of 22.6 kg (IQR: 19.2–26.6) and a median Fmean2 of 22.3 kg (IQR: 20.0–28.1) (*P* = .109). Fallers showed a slight decrease from 14.3 kg (IQR: 11.9–17.8) to 13.7 kg (IQR: 11.0–19.5), although this change was not statistically significant within the group (*P* = .073). However, the differences between fallers and non-fallers were statistically significant (*P* < .001). Regarding fatigue, non-fallers maintained a stable fatigue ratio of 1.2 (IQR: 1.0–1.2) at both time points (*P* = .299). Fallers had an initial Fatigue Ratio of 1.4 (IQR: 1.3–1.6), which increased significantly to 1.7 (IQR: 1.4–1.9) after 1 hour (*P* = .021). The differences between the groups were statistically significant (*P* < .001). Finally, the recovery ratio for non-fallers was 1.0 (IQR: 1.0–1.1), while fallers had a lower recovery ratio of 0.9 (IQR: 0.8–0.9), with statistically significant differences between the 2 groups (*P* < .001).

**Table 3.** Results of different functional health variables in handgrip strength (Fmax, Fmean, fatigue and recovery) in two rounds.

Variables	Non-fallers	*P*-value within groups	Fallers	*P*-value within groups	*P*-value between fallers/non-fallers
Fmax kg standard, median (IQR)	24.6 (22.3–31.1)	<.001	14.9 (12.1–19.7)	<.001	<.001
Fmax1 kg, median (IQR)	24.1 (21.2–30.9)		15.4 (12.7–19.8)		<.001
Fmax2 kg, median (IQR)	25.4 (21.7–32.1)		13.8 (11.8–19.0)		<.001
Fmean1 kg, median (IQR)	22.6 (19.2–26.6)	.109	14.3 (11.9–17.8)	.073	<.001
Fmean2 kg, median (IQR)	22.3 (20.0–28.1)		13.7 (11.0–19.5)		<.001
Fatigue Ratio1 kg, median (IQR)	1.2 (1.0–1.2)	.299	1.4 (1.3–1.6)	.021	<.001
Fatigue Ratio2 kg, median (IQR)	1.2 (1.1–1.3)		1.7 (1.4–1.9)		<.001
Recovery Ratio kg, median (IQR)	1.0 (1.0–1.1)		0.9 (0.8–0.9)		<.001

Note: The Wilcoxon signed-rank test was used for paired measurements (Round 1 and Round 2). The Friedman test was used to compare repeated measurements within the same group (Fmax standard, Fmax1, Fmax2). The Kruskal–Wallis test was used to assess differences between independent groups (Non-Fallers vs. Fallers). Fmax **=** Maximum handgrip strength in one session (highest value from 10 repetitions. Fmean = Mean handgrip strength across 10 repetitions. Fatigue Ratio = Fmax/Fmean – higher values indicate greater fatigability. Recovery Ratio = Fmean2/Fmean1 – higher values indicate better recovery capacity.

Table 4 shows that the strongest correlations with retrospective falls in the last 12 months were found for Short Physical Performance Battery score (*ρ*  = −.62), Recovery Ratio (*ρ*  = −.61), Falls Efficacy Scale-International score (*ρ*  = .60), Fatigue Ratio 1 (*ρ*  = .60) and Fatigue Ratio 2 (*ρ*  = .57), all with *P* < .001. Similarly, the same variables showed strong correlations for prospective falls within 4 months: Short Physical Performance Battery score (*ρ*  = −.58), Recovery Ratio (*ρ*  = −.58), Falls Efficacy Scale-International score (*ρ*  = .56), Fatigue Ratio 1 (*ρ*  = .57) and Fatigue Ratio 2 (*ρ*  = .56), all with *P* < .001.

**Table 4.** Spearman's rank correlations between the functional health variables and the retrospective and prospective number of falls.

	Retrospective falls in the last 12 months		Prospective falls in 4 months	
Variables	*ρ* (95% CI)	*P*-value	*ρ* (95% CI)	*P*-value
Retrospective Falls			0.74 (0.67–0.79)	<.001
SPPB Score	−0.62 (−0.69 – −0.51)	<.001	−0.58 (−0.66 – −0.48)	<.001
FES-I Score	0.60 (0.51–0.68)	<.001	0.56 (0.47–0.65)	<.001
Recovery Ratio	−0.61 (−0.69 – −0.51)	<.001	−0.58 (−0.65 – −0.49)	<.001
Fatigue Ratio 1	0.60 (0.50–0.69)	<.001	0.57 (0.46–0.65)	<.001
Fatigue Ratio 2	0.57 (0.46–0.65)	<.001	0.56 (0.45–0.63)	<.001
PASE Score	−0.47 (−0.57 – −0.35)	<.001	−0.42 (−0.53 – −0.30)	<.001
AIS Score	0.43 (0.31–0.53)	<.001	0.32 (0.19–0.44)	<.001
Standard maximum handgrip strength	−0.42 (−0.53 – −0.30)	<.001	−0.34 (−0.46 – −0.22)	<.001
MFI Score	0.36 (0.23–0.47)	<.001	0.39 (0.27–0.50)	<.001

Note: SPPB = Short Physical Performance Battery, FES-I = Falls Efficacy Scale-International Version.

PASE = Physical Activity Scale for the Elderly, AIS = Athens Insomnia Scale, MFI = Multidimensional Fatigue Inventory, Fatigue Ratio 1 and 2= Fmax/Fmean (higher values indicate higher fatigability), Recovery Ratio = Fmean2/Fmean1 – higher values indicate better recovery, Standard maximum handgrip strength = Highest value from three maximal attempts.

Table 5 shows that in the univariate analysis, all variables were significantly associated with falls, both for the 12 months prior to the survey (retrospective) and for the 4 months following the survey (prospective). However, in the fully adjusted model, only a subset of variables retained significant associations.

**Table 5.** Associations between functional health variables and falls 12 months before and 4 months after the survey.

Falls in the last 4 months since the survey (prospective)			
Variables	Univariate Crude Model IRR (95% CI)	Multivariable Model 1 IRR (95% CI)	Multivariable Model 2 IRR (95% CI)
Retrospective Falls	1.65 (1.30–1.80)***	1.42 (1.23–1.73)***	1.39 (1.18–1.66)***
SPPB Score	0.68 (0.62–0.79)***	0.81 (0.65–0.90)**	0.84 (0.68–0.92)**
FES-I Score	1.34 (1.12–1.48)***	1.13 (1.05–1.24)**	1.12 (1.07–1.39)*
PASE Score	0.76 (0.70–0.90)**	0.91 (0.79–0.99)*	0.93 (0.83–1.06)
AIS Score	1.06 (1.03–1.11)**	1.03 (0.90–1.13)	1.03 (0.88–1.09)
MFI Score	1.04 (1.02–1.07)**	1.04 (0.92–1.15)	1.03 (0.90–1.11)
Standard max. HGS	0.88 (0.79–0.92)**	0.95 (0.84–1.11)	0.94 (0.87–1.09)
Fatigue Ratio 1	1.44 (1.21–1.58)***	1.21 (1.12–1.51)**	1.14 (1.09–1.41)**
Recovery Ratio	0.70 (0.64–0.84)***	0.87 (0.73–0.97)*	0.89 (0.77–1.01)
Falls in the 12 months before the survey (retrospective)			
Variables	Univariate Crude Model IRR (95% CI)	Multivariable Model 1 IRR (95% CI)	Multivariable Model 2 IRR (95% CI)
SPPB Score	0.67 (0.58–0.82)***	0.83 (0.75–0.89)**	0.81 (0.64–0.86)**
FES-I Score	1.38 (1.10–1.57)***	1.17 (1.10–1.46)*	1.15 (1.10–1.42)*
PASE Score	0.92 (0.82–0.97)**	0.93 (0.78–1.06)	0.94 (0.81–1.11)
AIS Score	1.15 (1.08–1.31)**	1.11 (0.93–1.20)	1.09 (0.91–1.17)
MFI Score	1.14 (1.07–1.25)*	1.01 (0.80–1.09)	1.02 (0.81–1.09)
Standard max. HGS	0.82 (0.74–0.91)**	0.89 (0.78–1.08)	0.92 (0.84–1.11)
Fatigue Ratio 1	1.48 (1.29–1.87)***	1.19 (1.14–1.51)**	1.18 (1.13–1.53)**
Recovery Ratio	0.69 (0.60–0.83)***	0.88 (0.72–0.96)*	0.85 (0.72–0.91)*

Note: *P* value * <.05; ** <.01; *** <.001. IRR = Incidence rate ratio, SPPB = Short Physical Performance Battery, FES-I.

Falls Efficacy Scale-International Version, PASE = Physical Activity Scale for the Elderly, AIS = Athens Insomnia Scale.

MFI = Multidimensional Fatigue Inventory, Fatigue Ratio 1 and 2 = Fmax/Fmean (higher values indicate higher fatigability), Recovery Ratio = Fmean2/Fmean1 – higher values indicate better recovery, Standard maximum handgrip strength = Highest value from three maximal attempts.

Model 1: adjusted for age and sex.

Model 2: adjusted for sex, age, the Charlson Comorbidity Index, Barthel Index, Mini Mental State Examination, and number of medications.

For prospective falls (within 4 months of the survey), previous falls showed the strongest association, with each previous fall being associated with a 39% increase in the rate of future falls (Incidence Rate Ratio = 1.39, 95% Confidence Interval: 1.18–1.66, *P* < 0.001). A 1 point increase in the Short Physical Performance Battery score was associated with a 16% decrease in the incidence of falls (95% Confidence Interval: 0.68–0.92, *P* < .01), while a 1 point increase in the Falls Efficacy Scale-International score was associated with a 12% increase in the rate of falls (Incidence Rate Ratio = 1.12, 95% Confidence Interval: 1.07–1.39, *P* < .01). Fatigue Ratio 1 showed a significant association, with a 1-unit increase associated with a 14% higher fall rate (95% Confidence Interval: 1.09–1.41, *P* < .01). However, the recovery ratio, which was significant in the univariate analysis, lost its significance in the fully adjusted model (Incidence Rate Ratio = 0.89, 95% Confidence Interval: 0.77–1.01, *P* = .073). Notably, both Recovery Ratio (Incidence Rate Ratio = 0.87, 95% Confidence Interval: 0.73–0.99, *P* = .041) and Physical Activity Scale for the Elderly score (Incidence Rate Ratio = 0.91 (0.79–0.99), *P* = .047), which were significant in model 1, lost significance in model 2.

For retrospective falls, the Short Physical Performance Battery score showed a strong association in the fully adjusted model, with a 1 point increase being associated with a 19% decrease in fall incidence (95% Confidence Interval: 0.64–0.86, *P* < .01). The Falls Efficacy Scale-International score remained significant with a 15% increase in fall incidence per point (95% Confidence Interval: 1.10–1.42, *P* < .021). Fatigue Ratio 1 was associated with an 18% higher fall rate per unit increase (95% Confidence Interval: 1.13–1.53, *P* < .01). The Recovery Ratio remained significant with a 15% decrease in fall incidence per unit increase (95% Confidence Interval: 0.72–0.91, *P* < .039). The Physical Activity Scale for the Elderly and Athens Insomnia Scale scores, which were significant in the univariate models, did not retain their significance in the fully adjusted models

## Discussion

Our study explored the link between repetitive handgrip strength, muscle fatigability, recovery, and fall risk in older adults. Fallers exhibited significantly lower maximum and mean handgrip strength, fatigue ratio, and recovery ratio than non-fallers. Both standard and repetitive handgrip strength measures (Fmax1 and Fmax2) were lower in fallers, supporting findings that reduced muscle strength increases fall risk in older adults. The significant group difference highlights that maximum handgrip strength can discriminate between higher and lower fall risk. Our study shows that standardised maximum handgrip strength can be effectively measured using the repetitive handgrip strength protocol, as indicated by the median values (quartiles 1–3): Fmax standard 24.6 kg (22.3–31.1), Fmax1 24.1 kg (21.2–30.9) and Fmax2 25.4 kg (21.7–32.1). The handgrip strength protocol offers insights into muscle fatigability and recovery capacity beyond standard measurements. Incorporating multiple maximal efforts captures dynamic changes in muscle performance, reflecting real-life activities that require repeated muscle use. This offers a more comprehensive understanding of muscle function and enhances fall risk assessment. Fallers showed significantly higher fatigue ratios and a notable increase over time, indicating greater muscle fatigability. In contrast, non-fallers maintained stable fatigue ratios, reflecting consistent performance. Additionally, fallers displayed reduced recovery capacity, suggesting both a faster onset of fatigue and impaired recovery. These findings align with existing evidence linking muscle fatigability and recovery capacity to fall risk in older adults. He et al.^1^ reported that fatigue was significantly associated with an increased risk of falls in individuals aged 75 years and older, mediated by reduced lower limb function and balance confidence. Their study focused on lower limb fatigability, but our findings extend this to upper limb function and highlight the value of repetitive handgrip strength assessments for fall risk evaluation. We found reduced neuromuscular efficiency in fall-prone individuals, aligning with increased neuromuscular inhibition in older adults at risk of falls.^32^ The relationship between muscle fatigability and fall risk involves several physiological mechanisms. At the neuromuscular level, fatigue involves both central and peripheral components. Central fatigue involves reduced neural drive from the motor cortex and reduced motor unit recruitment, while peripheral fatigue affects the contractile properties of muscle fibres and metabolic function. During sustained or repeated contractions, this results in reduced force production capacity and slower muscle activation and relaxation times.^33^ Additionally, fatigue impairs proprioceptive feedback and alters motor control strategies, particularly affecting older adults’ ability to maintain postural control and respond to imbalances, increasing fall risk.^34^ Furthermore, other studies^4,35^ suggested that peripheral fatigability and corticospinal inhibition are more pronounced in the upper limbs than lower, making them useful for assessing neuromuscular fatigability and recovery. Our study supports this suggestion by demonstrating that repetitive handgrip strength measurements can effectively detect significant differences in fatigability and recovery between fallers and non-fallers.

Our study expands current knowledge by showing that objectively measured fatigability and recovery rates predict fall risk more strongly than standard maximum handgrip strength or self-reported fatigue. Previous falls were the strongest predictor (*ρ* = .74). Moderate correlations were found for the Short Physical Performance Battery (*ρ* = –.62 retrospective, −.58 prospective), fatigue ratio (*ρ* = .60/.57), and recovery ratio (*ρ* = –.61/−.58). In contrast, standard handgrip strength (*ρ* = –0.42/−0.34) and the Multidimensional Fatigue Inventory (*ρ* = .36/.39) showed weaker associations. These patterns were confirmed in logistic regression. Previous falls remained the strongest predictor of prospective falls (IRR = 1.39, 95% CI: 1.18–1.66). The SPPB score significantly predicted both retrospective (IRR = 0.81, CI: 0.64–0.86) and prospective falls (IRR = 0.86, CI: 0.68–0.92). Fatigue Ratio 1 was consistently associated with both fall types (IRR = 1.20/1.14). The FES-I score also predicted retrospective (IRR = 1.15, CI: 1.10–1.42) and prospective falls (IRR = 1.12, CI: 1.07–1.39), underscoring the psychological dimension of fall risk. Notably, standard handgrip strength and MFI scores were not significant in adjusted models, suggesting that objective fatigability parameters outperform subjective fatigue perception and single-strength measurements in predicting falls.

The results confirm the potential benefit of repetitive handgrip strength measurements and a broader assessment of muscle functionality in predicting fall risk and informing preventive interventions. The relative predictive power of objective versus subjective measures of fatigability for falls is unclear, but remains a topic of debate for different outcomes. Our findings, which support the superior predictive power of objective measures of fatigability, are consistent with studies like Egerton et al.,^36^ who have shown stronger associations between objective measures and physical activity levels in older adults. However, studies suggest that subjective fatigue captures cognitive and emotional aspects better than objective measures.^37,38^ Martino et al.^38^ found that subjective fatigue was associated with motor performance under cognitive load, while Holtzer et al.^37^ showed that it correlated with brain oxygenation during demanding tasks, even when objective measures such as gait speed did not. This suggests that objective measures are more predictive of physical outcomes, while subjective assessments provide insight into cognitive and emotional fatigue, with both playing complementary roles. The comparable predictive power of our fatigability and recovery measures to the established Short Physical Performance Battery is worth noting. While studies have shown mixed results regarding the predictive value of the Short Physical Performance Battery for falls,^39–41^ our study suggests that the measures derived from the repetitive handgrip strength protocol provide equally valuable information. Interestingly, Fatigue Ratio 1, based on the initial 10 repetitions, demonstrated predictive accuracy comparable to the Short Physical Performance Battery, while requiring significantly less time. Although calculating the recovery ratio necessitates a second measurement after 1 hour, it provides additional insight into muscle recovery. Taken together, Fatigue Ratio 1 and the recovery ratio offer a similarly comprehensive view of muscle function as the Short Physical Performance Battery, outperforming standard handgrip strength alone. These results support the use of repetitive handgrip testing as a time-efficient and clinically valuable tool for fall risk assessment, allowing for flexible application depending on available time and clinical priorities.

This study has several limitations. Firstly, its cross-sectional design limits the ability to draw conclusions about causality or changes over time. In addition, the significantly higher proportion of females (88.4%) compared to males (11.6%) in the fall group, together with their older age (83.4 vs. 78.3 years), may have influenced our results. Due to biological factors such as smaller muscle fibre size, lower muscle mass and lower levels of anabolic hormones, females generally have lower handgrip strength than males.^42^ These sex differences become more pronounced with age, especially after the menopause.^43^ Therefore, the observed lower handgrip strength and higher fatigue rates in the fallers group may partly reflect these sex-specific muscle characteristics rather than fall risk alone, even after statistical adjustment. This demographic pattern, with more women (particularly older women) in the faller group, is a common observation in residential care settings,^44^ but must be taken into account when interpreting the differences in muscle strength and fatigability between fallers and non-fallers. Another limitation of this study is that although we used a clear definition of falls (unintentional events in which a person comes to rest on the ground), we recognise that fall risk itself is multifactorial. Although we focused on physical performance, fear of falling, muscle strength and fatigability, falls can also be influenced by other intrinsic factors and environmental conditions. Our study used objective records of falls from medical records and 3D sensor-based assessments. Future studies could investigate how different risk factors interact, while maintaining the same clear definition of a fall. In addition, the use of surface electromyography would have provided additional insight into electrical muscle activity during sustained contractions, leading to a better understanding of muscle health and its relationship to fatigability and recovery. In addition, the recruitment of participants from residential care homes may have introduced selection bias, as the sample may not fully represent the wider older adult population, particularly those living independently, thereby limiting the generalisability of the findings. Future studies may benefit from incorporating these additional measurement techniques, recruiting a larger and more diverse sample of older adults, and employing a longitudinal design to further elucidate the complex relationships between muscle fatigability, recovery and fall risk in the older population.

**Clinical messages**

- Our findings suggest that fatigability and recovery ratios from repetitive handgrip strength assessments may provide more sensitive indicators of fall risk than maximum handgrip strength alone.
- Repetitive handgrip strength protocols offer dynamic insights into neuromuscular performance and could help identify individuals at higher fall risk.
- While these objective measurements show promise for clinical application, a comprehensive fall risk assessment must integrate multiple risk factors, including physical performance, balance, and environmental influences.
- Combining objective measures (handgrip strength parameters, SPPB) with subjective assessments (Falls Efficacy Scale) may enhance fall risk evaluation, though further validation in diverse populations is warranted.

## Supplemental Material

sj-docx-1-cre-10.1177_02692155251355881 - Supplemental material for Fatigue and recovery assessed by repetitive handgrip strength measurement as predictors of fall risk in older adults: A cross-sectional study Supplemental material, sj-docx-1-cre-10.1177_02692155251355881 for Fatigue and recovery assessed by repetitive handgrip strength measurement as predictors of fall risk in older adults: A cross-sectional study by Ali Kapan, Milos Ristic, Richard Felsinger and Thomas Waldhoer in Clinical Rehabilitation
