# Supplementary material for: Fatigue and recovery assessed by repetitive handgrip strength measurement as predictors of fall risk in older adults: A cross-sectional study
Source: Clin Rehabil. 2025 Jun 30;39(8):1116–29. doi: 10.1177/02692155251355881 (PMC12290228; doi:10.1177/02692155251355881)
Supplement: sj-docx-1-cre-10.1177_02692155251355881 - Supplemental material for Fatigue and recovery assessed by repetitive handgrip strength measurement as predictors of fall risk in older adults: A cross-sectional study [file sj-docx-1-cre-10.1177_02692155251355881.docx]

PLUS

STROBE Statement—Checklist of items that should be included in reports of cross-sectional studies

| **Item No** | **Recommendation** | **Page No** |
| --- | --- | --- |
| 1 (a) | Indicate the study’s design with a commonly used term in the title or the abstract | Title; Abstract |
| 1 (b) | Provide in the abstract an informative and balanced summary of what was done and what was found | Abstract |
| 2 | Explain the scientific background and rationale for the investigation being reported | Background: pp. 2–3 |
| 3 | State specific objectives including any prespecified hypotheses | Objectives: p. 3 |
| 4 | Present key elements of study design early in the paper | Study Design: p. 3 |
| 5 | Describe the setting, locations, and relevant dates, including periods of recruitment and data collection | Methods: pp. 3–6 |
| 6 (a) | Give the eligibility criteria and the sources and methods of selection of participants | Participants: p. 4 |
| 6 (b) | Describe methods of follow-up | Not applicable (cross-sectional) |
| 7 | Clearly define all outcomes, exposures, predictors, potential confounders, and effect modifiers | pp. 4–6 |
| 8* | For each variable of interest give sources of data and details of methods of assessment (measurement) | Measurements: pp. 4–6 |
| 9 | Describe any efforts to address potential sources of bias | Methods: pp. 4–6 |
| 10 | Explain how the study size was arrived at | Methods: p. 4 |
| 11 | Explain how quantitative variables were handled in the analyses. Describe groupings if applicable | Statistics: pp. 6–7 |
| 12 (a) | Describe all statistical methods, including those used to control for confounding | Statistics: pp. 6–7 |
| 12 (b) | Describe any methods used to examine subgroups and interactions | Statistics: p. 7 |
| 12 (c) | Explain how missing data were addressed | No missing data |
| 12 (d) | If applicable, describe analytical methods taking account of sampling strategy | Not applicable |
| 12 (e) | Describe any sensitivity analyses | Not applicable (exploratory) |
| 13 (a) | Report numbers of individuals at each stage of the study | Participants: p. 4 |
| 13 (b) | Give reasons for non-participation at each stage | Not explicitly described |
| 13 (c) | Consider use of a flow diagram | Not included |
| 14 (a) | Give characteristics of study participants (e.g. demographic, clinical, social) and info on exposures/confounders | Results: pp. 7–8 (Table 2) |
| 14 (b) | Indicate number of participants with missing data for each variable of interest | No missing data |
| 15* | Report numbers of outcome events or summary measures | Results: pp. 7–8 |
| 16 (a) | Give unadjusted and, if applicable, adjusted estimates with precision (e.g. 95% CI). Clarify adjusted confounders | Results: pp. 7–8 |
| 16 (b) | Report category boundaries when continuous variables were categorized | Not applicable |
| 16 (c) | Translate estimates of relative risk into absolute risk for meaningful time period (if relevant) | Not applicable |
| 17 | Report other analyses done (e.g., subgroup or sensitivity analyses) | Not applicable (exploratory) |
| 18 | Summarise key results with reference to study objectives | Discussion: pp. 9–10 |
| 19 | Discuss limitations of the study, including sources of potential bias and imprecision | Limitations: p. 12 |
| 20 | Provide cautious interpretation of results in context of objectives, limitations, and other evidence | Conclusions: p. 12 |
| 21 | Discuss generalisability (external validity) of study results | Limitations: p. 12 |
| 22 | Give source of funding and the role of funders | Funding: p. 12 |

*Give information separately for exposed and unexposed groups.

**Note:** An Explanation and Elaboration article discusses each checklist item and gives methodological background and published examples of transparent reporting. The STROBE checklist is best used in conjunction with this article (freely available on the Web sites of PLoS Medicine at http://www.plosmedicine.org/, Annals of Internal Medicine at http://www.annals.org/, and Epidemiology at http://www.epidem.com/). Information on the STROBE Initiative is available at www.strobe-statement.org.
